# Patellar Tendon Rupture after Lateral Release without Predisposing Systemic Disease or Steroid Use

**DOI:** 10.1155/2015/215796

**Published:** 2015-04-15

**Authors:** S. De Giorgi, A. Notarnicola, G. Vicenti, B. Moretti

**Affiliations:** Department of Basic Medical Science, Neuroscience and Sensory Organs, University of Bari, Piazza G. Cesare 11, 70124 Bari, Italy

## Abstract

Arthroscopic technique for lateral release is the most widely used procedure for the correction of recurrent dislocations of the patella. In the relevant literature, several complications of lateral release are described, but the spontaneous patellar tendon rupture has never been suggested as a possible complication of this surgical procedure. Patellar tendon rupture is a rather infrequent and often unilateral lesion. Nevertheless, in case of systemic diseases (LES, rheumatoid arthritis, and chronic renal insufficiency) that can weaken collagen structures, bilateral patellar tendon ruptures are described. We report a case of a 24-year-old girl with spontaneous rupture of patellar tendon who, at the age of 16, underwent an arthroscopic lateral release for recurrent dislocation of the patella. This is the first case of described spontaneous patellar tendon rupture that occurred some years after an arthroscopic lateral release.

## 1. Introduction

Patellar tendon ruptures are often associated with lupus erythematosus, rheumatoid arthritis, chronic renal disease, or chronic use of corticosteroids. Bilateral patellar tendon ruptures are extremely rare and have only been documented in some case series [1, 2].

In the present report we describe a case of unilateral spontaneous rupture of the patellar tendon in the absence of concomitant local or systemic diseases or steroid use.

## 2. Case Report

**Figure 1:**
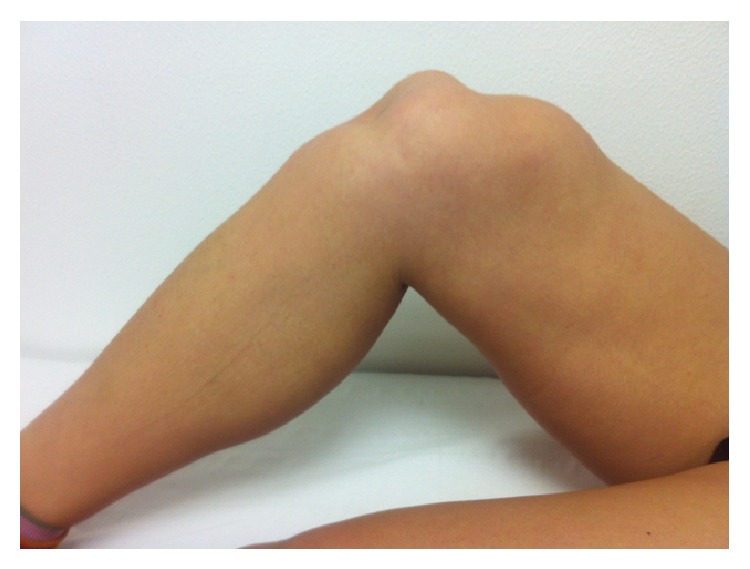
Lack of the normal anatomy of the right knee. The femoral condyles can be palpated.

**Figure 2:**
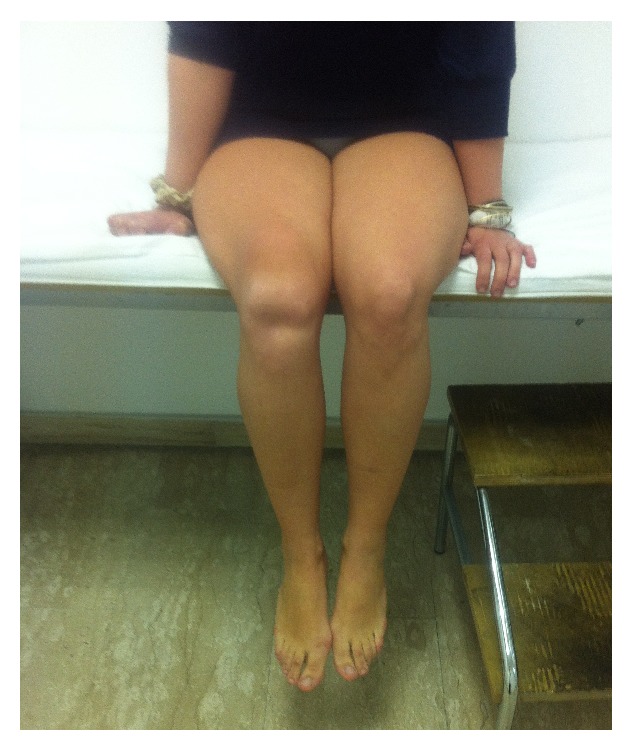
Active extension of the right knee was not possible although passive extension was complete.

**Figure 3:**
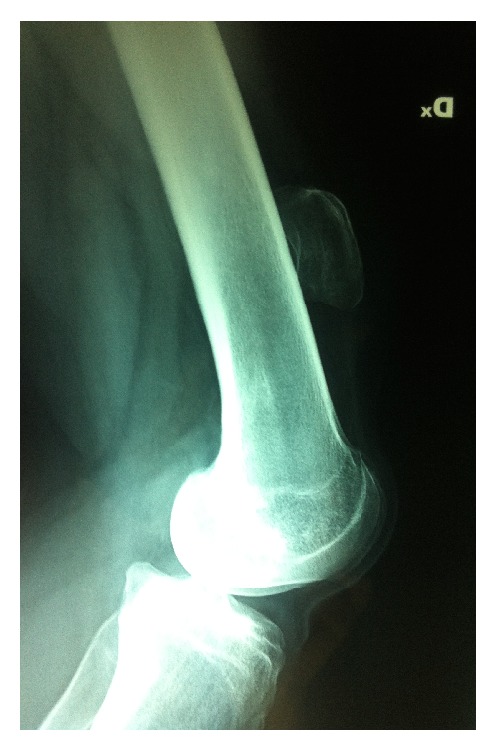
The LL X-ray of the knee showed no fractures, but an evident proximal dislocation of the patella and some calcific deposits on its inferior pole.

**Figure 4:**
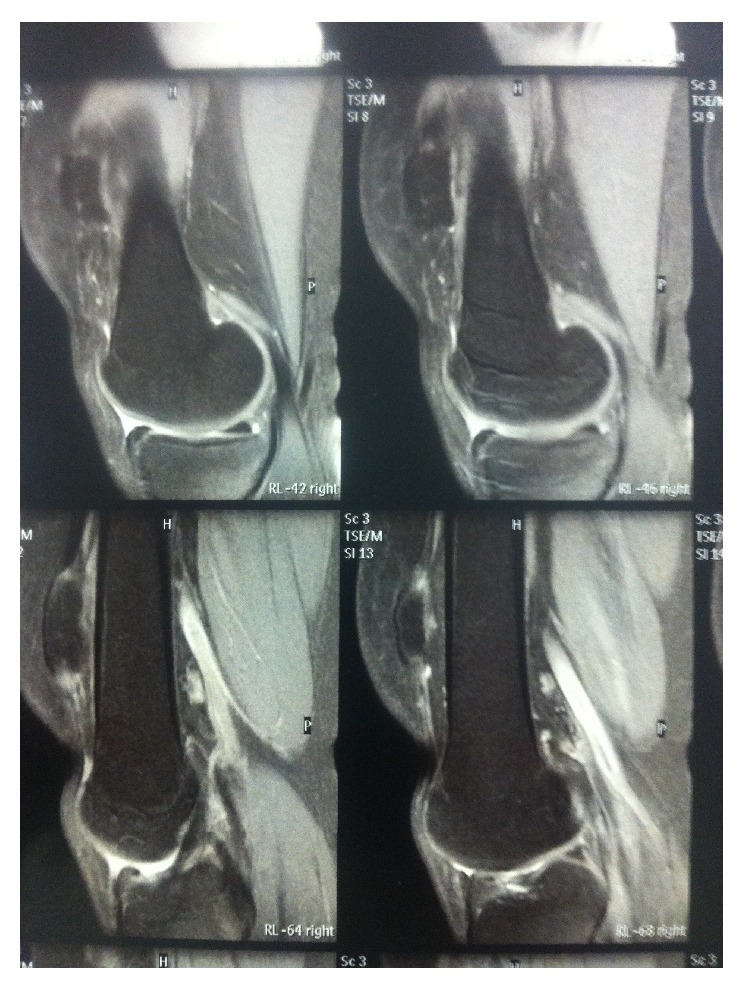
IRM of the knee showed a rupture of patellar tendon near the osteotendinous junction under the inferior pole of the patella.

We describe the case of a 24-year-old girl who, at the age of 16, underwent a lateral release arthroscopic surgery of the right knee for recurrent dislocations of the patella. She was asymptomatic for two years; then she reported a new dislocation of the patella that she reduced on her own. She was hospitalized and conservatively treated for the pain and swelling of the knee and then continued with physiotherapy for some months. After 6 years, without any new injury, she came to our hospital for a consultation, presenting a lack of the normal anatomy of the right knee (Figure 1). Her active extension of the right knee was not possible although passive extension was complete (Figure 2). The patient's history revealed neither obvious systemic disease nor any use of corticosteroids, tobacco, alcohol, or illicit drugs. The patient could walk or bear weight on both legs and also climb the stairs, but she was not able to actively extend the right knee. During clinical examination, there were no visible skin lesions around the knee whereas a gap inferior to the right patella was visible, through which femoral condyles could be palpated (Figures 1 and 2); meniscal and laxity tests were negative and neurological examination was normal. The AP and LL X-ray of the knee showed no fractures, but an evident proximal dislocation of the patella and some calcific deposits on its inferior pole (Figure 3). IRM of the knee showed a rupture of patellar tendon near the osteotendinous junction under the inferior pole of the patella (Figure 4). The laboratory exams were normal as well as immunologic tests with antibodies examination; only an increase in TSH was found.

The patient did not accept our suggestion for a surgical approach because she did not complain about important functional deficits.

## 3. Discussion

The patellar tendon normally has a strong structure. Some biomechanical studies reported that a force of 17.5 times the body weight is necessary [3] to cause the injury of a normal tendon, while on climbing stairs a force of 3.3 times the body weight is applied on the tendon [4].

The acute rupture is due to a contraction of the quadriceps muscle against a fixed structure or to a sudden increase in the loading on the patient, against a quadriceps in active contraction; in both cases there is an eccentric contraction of muscle fibers with a muscular stretching during the contraction.

Spontaneous rupture of patellar tendon is an infrequent disease. The exact epidemiology is not really known because of the lack of patients that undergo examination and that cannot be perfectly identified and treated. The bilateral patellar tendon rupture is even less frequent and only a few cases were described in the literature [1, 2, 5, 6]. A patellar tendon rupture usually occurs after a degeneration of the tendon in patients with systemic diseases or in subjects with previous intra-articular corticosteroid infiltrations [6]. Hypoxic and calcific tendinopathies are described, as well as mucoid degeneration and lipomatous metaplasia [7]. Some diseases can cause anomalies of the tendon such as lupus systemic erythematosus, diabetes, rheumatologic diseases, chronic renal failure, hyperparathyroidism, and local and systemic use of corticosteroids [1, 7, 8]. In our case report, none of these diseases was present.

Inflammatory problems, collagen necrosis, and amyloid deposits can weaken the tendon structure, thus becoming predisposing factors of the injury. Frequent tenosynovitis and microtraumas can produce a local stress with progressive tendon degeneration. Spontaneous tendon injuries are often preceded by histopathologic degenerations that can compromise the tendon integrity [7] and produce a tendon failure. This can occur after some surgical procedures that alter the extensor mechanism of the knee, such as total knee prosthesis or ACL reconstruction with patellar tendon graft [9].

Spontaneous rupture of patellar tendon can occur also after a bursectomy procedure [10].

In our case, the previous lateral release and the lack of new injuries and of systemic diseases led us to think of a possible consequence of the previous surgical procedure. The young patient had undergone a lateral release for recurrent dislocation of the patella 8 years before. Given the absence of immunologic and systemic diseases, we can presume a consequentiality in the two events, even after some years. Many authors reported successful short-term results after a lateral release. Aglietti et al. [11] compared three different options of treatment in recurrent dislocations of the patella and reported the worst long-term results with 35% of recurrence in the lateral release group in a retrospective study. Dainer et al. [12] agree with Aglietti et al. and suggest that lateral release has only a diagnostic value and cannot permanently solve the related problems. Kolowich et al. [13], in a similar study, reported a high rate of poor results in the lateral release with recurrence of instability and pain. Fulkerson suggests the use of the lateral release as an added procedure after proximal realignment of the patella and reconstruction of the MPFL (medial retinaculum, medial patellofemoral ligament) [14].

Lateral release is often a quick procedure, performed arthroscopically. Nevertheless some complications can occur, such as excessive bleeding and subcutaneous lesions [15]. The extension and direction of the lateral release can predispose to complications. Several authors underline that the incision of the retinaculum must include the insertion of the lateral vastus, determining the necessity to extend proximally for 6–8 cm on the medial edge of the musculotendinous junction. This aggressive approach can produce the weakness of tendon and predispose it to late failure.

Our case report underlines that it is mandatory to avoid extensive arthroscopic lateral release in order to minimize patellar tendon degeneration. Furthermore, patients should be informed of a potential risk of the arthroscopic lateral release, as the case presented in this paper.

## 4. Conclusion

In conclusion, we report a rare case of spontaneous patellar tendon rupture in the absence of systemic or local disease or steroid usage. Recurrent microtraumas for daily activity and previous surgery for extended lateral release may have contributed to degenerative changes and late failure of patellar tendon.
